# Some Mushrooms are Hard to Digest: Gastrostomy Tube Exchange

**DOI:** 10.12659/PJR.902203

**Published:** 2017-07-19

**Authors:** Nishant Gupta, Pradeep Goyal, Itisha Bansal, Kusum Hooda, Yogesh Kumar, Gregory Bearden

**Affiliations:** 1 Department of Radiology and Imaging, Saint Vincent’s Medical Center, Bridgeport, CT, U.S.A; 2 Department of Anesthesiology, New York Methodist Hospital, Brooklyn, NY, U.S.A.; 3 Department of Radiology, Yale New Haven Health at Bridgeport Hospital, Bridgeport, CT, U.S.A.; 4 Department of Surgery, Baptist Health System Inc., Birmingham, AL, U.S.A.

**Keywords:** Endoscopy, Gastrointestinal, Enteral Nutrition, Gastrostomy

## Abstract

**Background:**

Percutaneous endoscopic gastrostomy (PEG) is an effective and safe mode of enteral nutrition for patients needing chronic enteric nutritional support. Exchanging PEG tubes may result in complications due to inexperience as well as due to lack of protocol.

**Case Report:**

We encountered a 73 year-old female with unnoticed, accidently detached portion of the internal bumper of a PEG tube in the gastric lumen after a challenging gastrostomy tube exchange.

**Conclusions:**

This case report discusses the complications associated with gastrostomy tube exchange and proposes a planned protocol for successful gastrostomy tube exchange.

## Background

Percutaneous endoscopic gastrostomy (PEG) tube is a well-accepted route of enteral nutrition in chronically malnourished patients with impaired swallowing [1]. Two methods are commonly employed to remove PEG. One method entails a complete removal of the PEG and its internal bumper (mushroom) through the anterior abdominal wall. If a complete forward extraction is not possible, PEG tube can be removed by cutting the tube close to the skin, and then the inner bumper is either left inside the bowel to pass spontaneously (“cut and push” method) or retrieved endoscopically. We describe a patient whose long-term, bumper-retained PEG tube was removed through the anterior abdominal wall with difficulties and exchanged for a balloon-retained PEG tube at a skilled nursing facility. However, the internal bumper of the PEG tube was detached in the gastric lumen accidently, which was subsequently retrieved endoscopically. If such detached internal bumpers are left *in situ* allowing them to transit to the GI tract, it may lead to various complications, including intestinal obstruction and/or perforation or even death [2–5].

## Case Report

**Figure 1:**
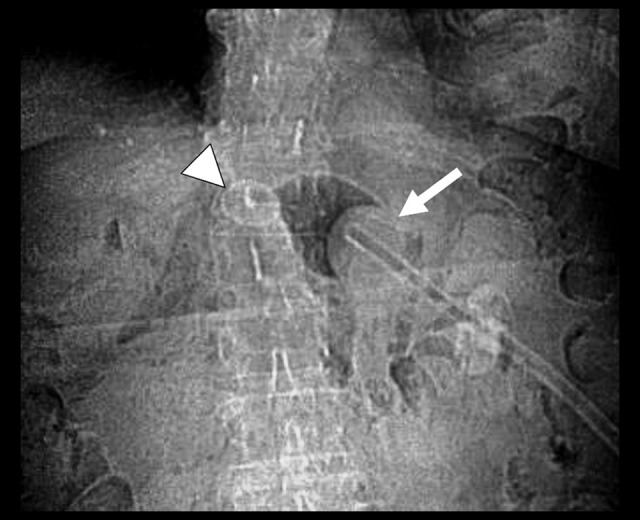
Abdominal radiograph demonstrates a round, radiodense disc in the mid abdomen, not connected to the balloon-retained PEG tube.

**Figure 2:**
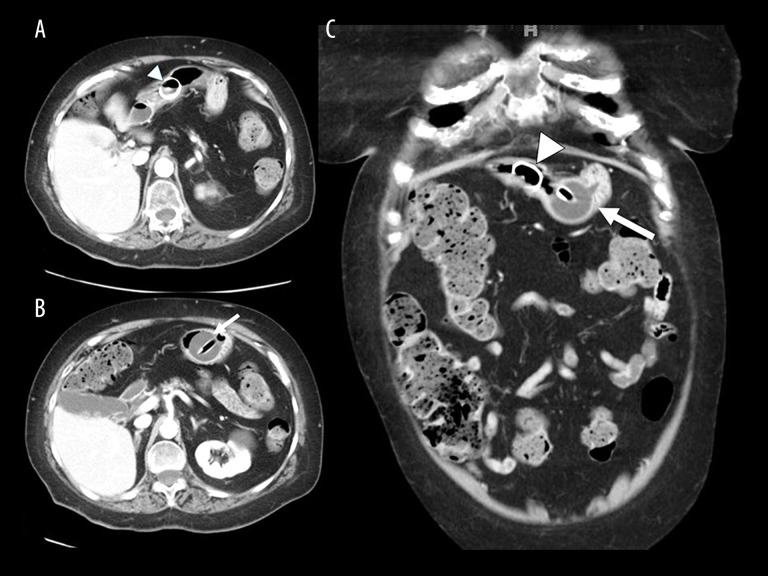
Axial and coronal CT images of the abdomen and pelvis demonstrate a round, radiodense disc in the gastric lumen (arrowhead in **A** and **C**), not connected to the balloon-retained PEG tube (long arrow in **B** and **C**).

**Figure 3:**
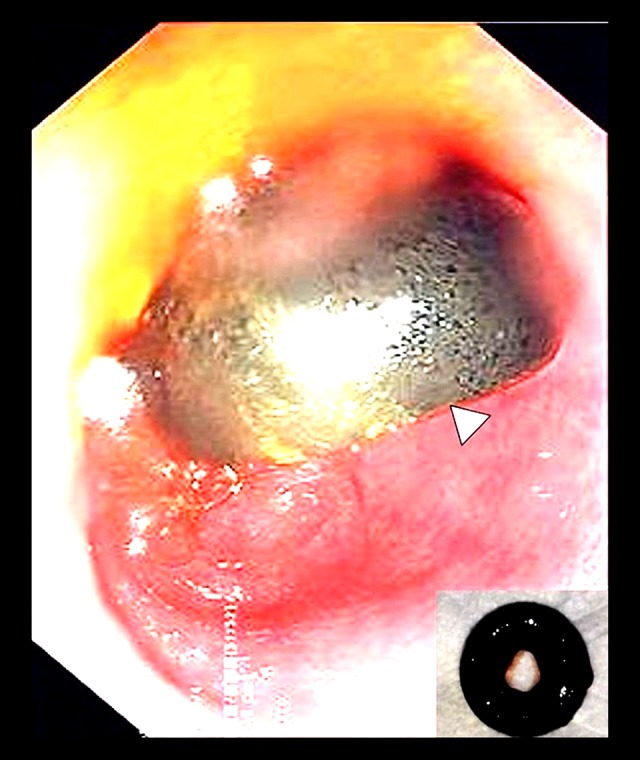
Endoscopy image demonstrates a disc-like structure, representing the detached internal bumper of the PEG tube in the lumen of stomach with surrounding inflammatory changes in the gastric mucosa. Retrieved retained internal bumper [inset].

A 73-year-old female with chronic malnutrition presented with one-day history of small amount of blood ooze from the PEG skin entry site. Physical examination revealed healthy granulation tissue around the PEG tube with minimal ooze. The patient also complained of non-specific pain in the upper abdomen for 1 month, soon after gastrostomy exchange outside her nursing facility. The prior mushroom-retained PEG tube was exchanged for a new balloon-retained gastrostomy tube. Perhaps, the removed PEG tube was not thoroughly evaluated for any missing parts and no follow-up abdominal radiograph was performed as per their protocol. An abdominal radiograph and CT scan of the abdomen and pelvis revealed a round, radiodense disc in the gastric lumen that was not connected to the balloon-retained PEG tube (Figures 1, 2). The balloon-retained PEG tube was intact and was in an appropriate position. A diagnosis of accidently detached intra-gastric internal bumper (mushroom) of the PEG tube was made. These findings were discussed with the patient who opted for an endoscopic retrieval of the retained internal bumper. At endoscopy, a dark-green to black disc-like structure, representing the detached internal bumper of the PEG tube, was identified in the lumen of stomach with surrounding inflammatory changes in the gastric mucosa (Figure 3). Moreover, the replaced balloon-retained gastrostomy tube was intact. After multiple attempts, the retained internal bumper/mushroom was retrieved endoscopically, using a foreign body retrieval forceps (Figure 3-inset).

## Discussion

PEG tube placement is a well-accepted route of enteral nutrition in chronically malnourished patients with impaired swallowing [1]. The first description of the PEG technique using a Gauderer-Ponsky tube (CR Bard Incorporated, Tewksbury, MA) comes from 1958, when Gauderer et al. reported it in the pediatric population [6]. Often, PEG tube needs replacement due to occlusion of the lumen or other factors. Replacement of gastrostomy tube is generally considered as a safe and simple procedure, even though various well-known complications include interruption of the continuity of the tract, incorrect tube placement in the peritoneum, peritonitis due to leakage of gastric contents and even death [2,5,7]. Anterograde extraction and the “cut and push” technique are the two commonly performed methods of PEG tube removal. In the “cut and push” method, the inner bumper is left inside the bowel to pass spontaneously and is monitored through serial abdominal radiographs at 7 and 14 days [3]. A number of published case series have shown the “cut and push” method to be quite safe with rare complications including intestinal obstruction and/or perforation or even death [3]. A limited literature review of published series recommend endoscopic removal of internal bumper after “cut and push” methods, especially in patients with risk factors for a retained internal bumper which include GI motility disorders, malrotation syndromes or prior abdominal surgery [3].

We encountered a rare complication of anterograde extraction of PEG tube with accidently detached and retained intra-gastric internal bumper/mushroom. This may be due to excessive pulling force during PEG tube removal, in addition to the loss of pliability of silicone in the internal bumper due to prolonged action of the gastric contents, leading to difficulty in delivering through the skin site opening [5]. Loss of pliability may also result in difficult retrieval of the retained internal bumper endoscopically, in addition to the narrow and vertical orientation of the gastroesophageal junction.

Different methods have been described in the literature for gastrostomy tube exchange; however, there are no consensus guidelines, which results in a wide variation of replacement and removal techniques [2,8,9].We recommend that each institution should establish an optimal protocol to prevent complications. The principles that govern any gastrostomy tube exchange are as follows; firstly, the duration of time for which the gastrostomy tube has been present in the body is of utmost importance, as it corresponds to gastrostomy tract maturation. Most PEG tube tracts start maturing at 1–2 weeks and are well-delineated within 4–6 weeks; however, the maturation time is also dependent on the nutritional status of the patient. The literature recommends no replacement of the gastrostomy tube without endoscopic visualization within 8 weeks of its placement [2]. Secondly, minimal insertional force should be applied at the time of replacement, as the tract between the stomach and skin is friable and can disrupt, complicating the tube displacement in the peritoneum. Thirdly, the removed PEG tube should be thoroughly evaluated for any missing parts. Fourthly, the mushroom-retained PEG tube should always be exchanged with a balloon-retained gastrostomy tube. Lastly, confirmation of tube position should be performed either radiographically or endoscopically. Injection of water-soluble contrast media through the tube under fluoroscopic guidance is the gold standard for confirmation [10]. Additionally, physicians should be aware that many PEG feeding tubes are not designed for percutaneous removal and should be removed endoscopically.

## Conclusions

This article puts an emphasis on a potential, dreaded complication associated with a simple procedure. This case represents a perfect example of how a simple procedure, such as gastrostomy tube exchange, may lead to disasters if not performed strategically. While awaiting consensus guidelines, we recommend that every institution should establish an optimal protocol for gastrostomy tube exchanges to minimize complications.
